# Citrullinated Fibrinogen Renders Clots Mechanically Less Stable, but Lysis-Resistant

**DOI:** 10.1161/CIRCRESAHA.121.319061

**Published:** 2021-05-26

**Authors:** Imre Varjú, Nicoletta Sorvillo, Deya Cherpokova, Ádám Z. Farkas, Veronika J. Farkas, Erzsébet Komorowicz, Tímea Feller, Balázs Kiss, Miklós Z. Kellermayer, László Szabó, András Wacha, Attila Bóta, Colin Longstaff, Denisa D. Wagner, Krasimir Kolev

**Affiliations:** 1 Program in Cellular and Molecular Medicine, Boston Children’s Hospital, MA (I.V., N.S., D.C., D.D.W.).; 2 Department of Pediatrics, Harvard Medical School, Boston, MA (I.V., N.S., D.C., D.D.W.).; 3 Department of Biochemistry (I.V., Á.Z.F., V.J.F., E.K., L.S., K.K.), Semmelweis University, Budapest, Hungary.; 4 Department of Biophysics and Radiation Biology (T.F., B.K., M.Z.K.), Semmelweis University, Budapest, Hungary.; 5 Department of Sociomedical Sciences, Mailman School of Public Health, Columbia University, NY (I.V.).; 6 Department of Functional and Structural Materials (L.S.), Institute of Materials and Environmental Chemistry, Research Centre for Natural Sciences, Hungarian Academy of Sciences, Budapest, Hungary.; 7 Biological Nanochemistry Research Group (A.W., A.B.), Institute of Materials and Environmental Chemistry, Research Centre for Natural Sciences, Hungarian Academy of Sciences, Budapest, Hungary.; 8 National Institute for Biological Standards and Control, South Mimms, United Kingdom (C.L.).; 9 Division of Hematology/Oncology, Boston Children’s Hospital, MA (D.D.W.).

**Keywords:** citrullination, fibrin, fibrinolysis, plasminogen, thrombosis, thromboelastography

**Meet the First Author, see p 220**

Clot stability, the resistance to mechanical stress and fibrinolytic dissolution, might affect the clinical behavior of deep vein thrombi and the success of thrombolysis or pharmacomechanical thrombectomy. Clot stability is sensitive to chemical modifications of the underlying fibrin matrix and its precursor fibrinogen. Citrullination is a posttranslational modification of peptidyl-arginine by PADs (peptidyl-arginine-deiminases).^1^ Both PAD2 and 4—proven to catalyze fibrinogen citrullination—have been detected in human serum and may be released from neutrophils during—or independently of—the formation of the highly procoagulant neutrophil extracellular traps.^1^ However, (1) the effect of citrullinated fibrinogen (CitFg) on clot stability is poorly understood and (2) no study has investigated CitFg in intravascular thrombi. Here, we provide evidence that citrullination greatly impacts the stability of human model thrombi and that CitFg is present in a murine analogue of human deep vein thrombi.

To incorporate CitFg in model thrombi, we incubated human fibrinogen with PAD4 in 100 mmol/L TRIS-HCl 10 mmol/L CaCl_2_ 1 mmol/L DTT pH 7.5 at 37 °C (concentrations and incubation times were adjusted for different experiments). PAD4 activity was halted with 60 mg/L Cl-amidine (in controls immediately at 0 hours).

We studied the mechanical properties of clots using atomic-force microscopy-based nano-thromboelastography^2^ as well as oscillation rheometry.^3^ In nano-thromboelastography, the forming fibrin deflects the vertically traveling cantilever immersed into the clot (6 µmol/L fibrinogen incubated with 0.25 mg/L PAD4 for 0/3 hours, clotted with 10 nmol/L thrombin). Fibrin strength is calculated from the deflection as the force difference (ΔF) between the onset and end of cantilever pulls. ΔF was markedly decreased throughout the clotting of CitFg, and the ratio of times elapsed from start until the maximal slope of force-curves was 3.10±0.68 (3/0 hours citrullinated fibrin, n=3), indicating delayed clotting (Figure [A]). This was in line with electromechanical coagulometry results: 17 nmol/L thrombin yielded clotting times 37.6±2.6/77.8±6.9*/87.4±6.0** s with 4 µmol/L fibrinogen citrullinated with 0.4 mg/L PAD4 for 0/3/4 hours, respectively (n=7, Kruskal-Wallis test *P*=0.0002, *P=0.0152, **P=0.0017 versus 0 hours, Dunn-test; mean±SE of mean shown throughout, statistical analyses performed with GraphPad Prism 9.00).

**Figure.**
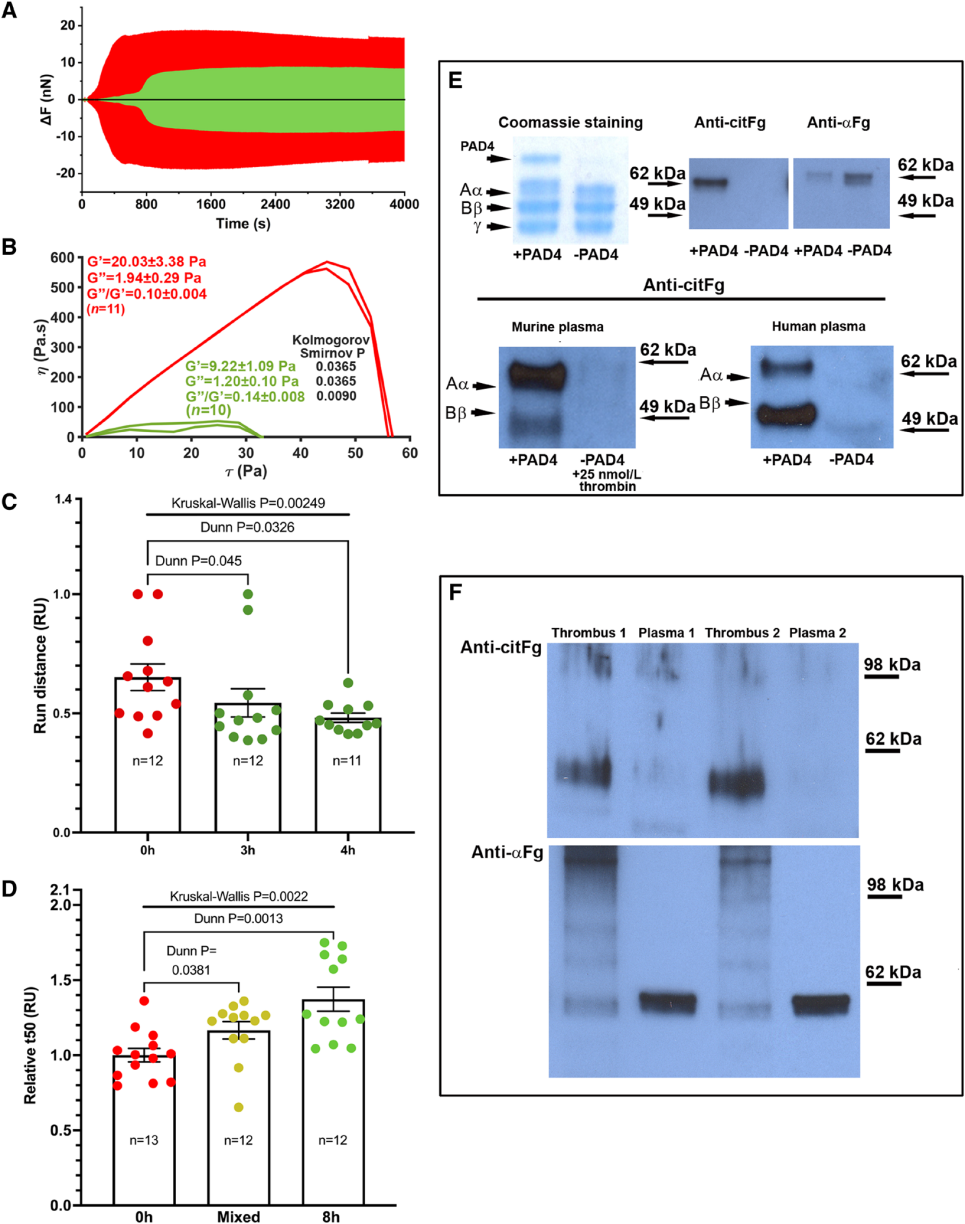
**Citrullinated fibrin(ogen): effects on mechanical (A and B) and fibrinolytic (C and D) stability, presence in murine samples (E and F).** Mean±SE of mean shown. Red/green: control/citrullinated. Animal work was approved by the Institutional Animal Care and Use Committee (No. 17-01-3308R). **A**, Nano-thromboelastography (nanoTEG). Fibrin strength during clotting.^2^ **B**, Microrheology. 0.015 oscillatory strain at 1 Hz imposed 2 min after clotting initiation.^3^ G′, G″: storage and loss moduli after 15 min clotting. Curves: fibrin viscosity (η) vs increasing shear stress (τ) postclotting.^3^ Factor XIIIa-inhibitor ZED1301 added at 20 µmol/L. **C**, Photoscanning. tPA (tissue-type plasminogen activator)-induced lysis of plasma clots spiked with fibrinogen (citrullinated for indicated time). Lysis-front run distances after 45 min were normalized for channel length (relative units, RU). **D**, Turbidimetry. Clotted fibrinogen (PAD4 [peptidyl-arginyl-deiminase-4]-treated for 0/8 h, or their 1:1 mixture) lysed with plasmin (1 µmol/L).^3^ Time until half-maximal turbidity at 340 nm (t50) was normalized using control t50 as reference. **E**/**F**, Polyacrylamide-gelelectrophoresis, western blot. 0.05 g/L PAD4 applied for 3h where indicated. **E**, **upper** row: ≈1 kDa shift in the apparent size of Aα chain upon citrullination of 0.3 μmol/L murine fibrinogen, resembling the shift previously shown in citrullinated human fibrinogen. **E**, **lower** row: citrullination patterns are similar in hirudin-anticoagulated recalcified murine vs human plasma in vitro, absent from native plasma (±clotted with thrombin). Note: molecular weights of murine fibrinogen chains are lower than human. **F**, Homogenized inferior vena cava (IVC) thrombi and corresponding plasma 48 h postligation. Citrullinated Aα chains detected by anticitrullinated fibrinogen (CitFg)/anti-alpha antibodies (**upper/lower**). To calculate normalized citrullination intensity, citrullinated Aα signals (**upper**) were normalized by citrullination nonspecific fibrinogen antibody signal intensity from stripped and reblotted membranes (not shown).

Assuming that the nano-thromboelastography cantilever behaves as a Hookean spring, the maximal *ΔF* is analogous with the maximal clot elasticity measured by conventional thromboelastometry.^2^ The ratio of maximal *ΔF* of 3/0 hours citrullinated fibrin was 0.52±0.08 (n=3), indicating reduced clot elasticity,^2^ in line with rheometry^3^ results of clots formed with 10 nmol/L thrombin and fibrinogen (8 µmol/L citrullinated with 0.6 mg/L PAD4). At the end of clotting, storage and loss moduli (G′, G″) stabilized at lower values in citrullinated clots, indicating decreased elastic and viscous properties, respectively, while the loss tangent (G″/G′) increased, revealing overall decreased plastic stability. Compared with control, flow-curves of citrullinated clots presented with markedly lower dynamic viscosity and a lack of strain hardening as increasing shear stress was applied (Figure [B]). Gel/fluid transition (marking clot disassembly^3^) occurred at 41.2±2.5/62.8±5.8 Pa in 3/0 hours citrullinated clots (n=10/11, *P*=0.0033, Kolmogorov-Smirnov test), indicating decreased resistance to shear stress upon citrullination.

Besides fibrin biomechanics, the persistence of thrombi also depends on their susceptibility to plasmin, a serine protease formed from plasminogen by plasminogen activators (eg, tPA [tissue-type plasminogen activator]). Therefore, we investigated the lysis of clotted recalcified human plasma spiked with 1 µmol/L fibrinogen (pretreated with 1.15 mg/L PAD4 for 0/3/4 hours) in transparent microfluid channels. Fibrin/fluid interface migration was followed by photoscanning after applying tPA (0.3 µmol/L).^3^ CitFg-supplemented clots lysed slower (Figure [C]). Similarly, turbidimetry showed decelerated plasmin-mediated degradation of clots formed with 15 nmol/L thrombin and 6 µmol/L fibrinogen (pretreated with 1.15 mg/L PAD4, Figure [D]).

To test the presence of CitFg in thrombi, we performed the inferior vena cava stenosis model in 8- to 10-week old wild-type C57BL/6J male mice. In this model, thrombus development (under restricted flow without direct endothelial damage) closely resembles human deep vein thrombi.^4^ First, we established that citrullinated murine fibrinogen shows a pattern similar to that of human fibrinogen on Western blot (Figure [E]). Next, we blotted processed inferior vena cava thrombi (samples were encoded upon blinded harvest), which showed a strong citrullination signal corresponding the Aα-chain (Figure [E] and [F]). Normalized citrullination intensity was 0.53±0.19 (n=6) within 1-3 h inferior vena cava ligation, with no statistically significant change at later timepoints (0.31±0.11 at 6-12 h, n=9; 0.49±0.14 at 24-48 h, n=7). In plasma from thrombus-bearing mice and in vitro clots generated thereof, CitFg was hardly detectable: normalized citrullination intensity was 0.04±0.02 for plasma; 0.04±0.01 for plasma clots (n=4, Kolmogorov-Smirnov test *P<0.05* when either is compared with any thrombus age group).

Here, we demonstrate that CitFg—previously identified in rheumatoid arthritis and atherosclerotic plaques, but not in intravascular clots—(1) markedly reduces clot stability and increases resistance to lysis in vitro and (2) is abundant in a murine analogue of human deep vein thrombi. Since microrheological parameters of clots are associated with thromboembolism recurrence,^5^ our findings warrant future studies to test how our proposed novel mechanism regulating clot stability translates into clinical characteristics of thrombi.

## Data availability

The data that support the findings of this study are available from the corresponding author upon reasonable request.

## Acknowledgments

We are grateful to Györgyi Oravecz, Krisztián Bálint, Long Chu, and Steve Cifuni for excellent technical assistance.

## Sources of Funding

This work was supported by the Higher Education Institutional Excellence Programme of the Ministry of Human Capacities in Hungary for the Molecular Biology thematic programme of Semmelweis University and STIA-2020-OTKA/137209 to K. Kolev, National Heart, Lung, and Blood Institute of the National Institutes of Health (grant R35 HL135765 to D.D. Wagner), the Hungarian National Research, Development and Innovation Office (NKFIH) No. PD124451 to A. Wacha and the Excellence Program BIONANO_GINOP-2.3.2-15-2016-00017 to A. Bóta. I. Varjú was supported by postdoctoral fellowships from the Hungarian Academy of Sciences, the Fulbright Commission as well as the Tempus Public Foundation, and a scholarship by the Rosztoczy Foundation. D. Cherpokova was supported by a postdoctoral fellowship of the German Research Foundation (CH 1734/1-1). A.Z. Farkas and V.J. Farkas received EFOP (Human Resource Development Operational Program) scholarship (EFOP-3.6.3-VEKOP-16-2017-00009).

## Disclosures

None.

## Supplementary Material


