# Supplementary material for: Citrullinated Fibrinogen Renders Clots Mechanically Less Stable, but Lysis-Resistant
Source: Circ Res. 2021 May 26;129(2):342–4. doi: 10.1161/CIRCRESAHA.121.319061 (PMC8260470; doi:10.1161/CIRCRESAHA.121.319061)
Supplement: Supplementary file 1 [file res-129-342-s001.pdf]

Major Resources Table

In order to allow validation and replication of experiments, all essential research materials listed in the Methods should be included in the Major Resources Table below. Authors are encouraged to use public repositories for protocols, data, code, and other materials and provide persistent identifiers and/or links to repositories when available. Authors may add or delete rows as needed.

Animals (in vivo studies)

Species	Vendor or Source	Background Strain	Sex	Persistent ID / URL
mouse	Jackson Laboratory (Bar Harbor, Maine)	C57BL/6J	male	www.jax.org

Antibodies

Target antigen	Vendor or Source	Catalog #	Working concentration	Lot # (preferred but not required)	Persistent ID / URL
fibrinogen (chain- and citrullination-nonspecific)	DAKO, Agilent Technologies, Santa Clara, CA	A0080	1:1,000		www.agilent.com
fibrinogen (alpha-chain specific)	Boster Bio, Pleasanton, CA	A00816-2	1:500		www.bosterbio.com
citrullinated fibrinogen (clone 1F11)	ModiQuest, Oss, The Netherlands	MQR2.101-100	1 mg/L		www.immunoprecise.com
mouse IgG (H+L)	Bio-Rad, Hercules, CA	170-6516	1:10,000		www.bio-rad.com
human IgG (H+L)	Bio-Rad, Hercules, CA	AHP1323P	1:10,000		www.bio-rad.com
rabbit IgG (H+L)	Bio-Rad, Hercules, CA	170-6515	1:10,000		www.bio-rad.com

Other

Description	Source / Repository	Persistent ID / URL
peptidyl-arginyl-deiminase-4 (PAD4, GST tagged human)	Sigma-Aldrich Kft, Budapest, HUngary	https://www.sigmaaldrich.com/catalog/product/sigma/srp5226?lang=hu&region=HU
Ac-(D)-Asp-MA-Nle-Nle-Leu-Pro-Trp-Pro-OH, "ZED1301" Site specific irreversible inhibitor of plasma transglutaminase (FXIII)	ZEDIRA GmbH, Darmstadt, Germany	https://zedira.com/

DOI [to be added]
